# Tablet App Based Dexterity Training in Multiple Sclerosis (TAD-MS): Research Protocol of a Randomized Controlled Trial

**DOI:** 10.3389/fneur.2019.00061

**Published:** 2019-02-11

**Authors:** Judith J. W. van Beek, Erwin E. H. van Wegen, Cleo D. Bol, Marc B. Rietberg, Christian P. Kamm, Tim Vanbellingen

**Affiliations:** ^1^Neurocenter, Lucerne Cantonal Hospital, Lucerne, Switzerland; ^2^Gerontechnology and Rehabilitation Group, University of Bern, Bern, Switzerland; ^3^Department of Rehabilitation Medicine, Amsterdam Movement Sciences, MS Center Amsterdam, Amsterdam University Medical Center VUmc, Amsterdam, Netherlands; ^4^Department of Neurology, Inselspital University Hospital Bern, University of Bern, Bern, Switzerland

**Keywords:** randomized controlled trial, app-based training, dexterity, multiple sclerosis, arm function in multiple sclerosis questionnaire

## Abstract

**Introduction:** Patients with Multiple Sclerosis exhibit disturbed dexterity, leading to difficulties in fine motor skills such as buttoning a T-shirt or hand-writing. Consequently, activities of daily living and quality of life are affected. The aim of the present study is to investigate the effectiveness of a tablet app-based home-based training intervention to improve dexterity in patients with Multiple Sclerosis.

**Methods:** An observer-blinded randomized controlled trial will be performed. Seventy patients with Multiple Sclerosis with self-reported difficulties in dexterity while executing activities of daily living will be recruited. After baseline assessment, participants are randomized to either an intervention group (*n* = 35) or control group (*n* = 35) by a computerized procedure. Blinded assessments will be done at baseline, post-intervention (after 4 weeks) and 12 weeks follow-up. The home-based intervention consists of a 4-week tablet app-based dexterity program. The app contains six dexterity games in which finger coordination, tapping, pinch grip is required. The control group will receive a Thera-band training program focused on strengthening the upper limb. The primary outcome is the Arm function of Multiple Sclerosis Questionnaire, a measure of patient-reported activities of daily living related dexterity. Secondary outcomes are dexterous function, hand strength, and quality of life.

**Discussion:** This study will evaluate the effects of tablet app-based training for dexterity in patients with Multiple Sclerosis. We hypothesize that a challenging app-based dexterity program will improve dexterity both in the short term and the long-term. The improved finger and hand functions are expected to generalize to improved activities of daily living and quality of life.

## Background

Multiple sclerosis (MS) is a chronic inflammatory disease of the central nervous system and the most common cause of non-traumatic disability in young adults (1–3). It is a heterogeneous disease, which is associated with long-term disability, leading to reduced Health related Quality of Life (HrQoL). While disease-modifying pharmacological therapies can decrease disease activity and progression, and symptomatic pharmacological treatments reduce complaints to a certain extent (3), patients with MS (PwMS) often still suffer from various neurological deficits during the course of their disease (1). Consequently, specific non-pharmacological rehabilitation is needed in order to further reduce disability and improve HrQoL (2, 4, 5). Evidence is accumulating that targeted rehabilitation drives neuroplasticity in MS (6).

Impaired dexterity is a frequently observed impairment, affecting up to 76% of PwMS (7). The different neurological deficits caused by MS, such as ataxia, spasticity, sensory-motor deficits, and apraxia may by themselves or in combination, impair dexterity (8, 9). Anatomically, it has been shown by Bonzano et al. (10) that reduced microstructural integrity of the corpus callosum, caused by the MS pathology, is associated with deficits in bimanual finger movements, therefore possibly explaining impaired dexterity (10). Consequently, PwMS report impairments in the performance of several activities of daily living (ADL), such as grooming, cooking, which may have a huge impact on the self-reported general health perception (11). Sometimes these problems are even associated with loss of work, and lack of social integration (12). Two systematic reviews by Lamers et al. highlighted the importance of a thorough standardized assessment and treatment of upper limb dysfunction in MS, more specifically impaired dexterity (4, 13). In a previous randomized controlled trial (RCT) we showed that a home-based unsupervised dexterity program can improve dexterity in MS (14). The program however did not allow for online monitoring of the patient's performance, meaning that no direct feedback could be given by the therapist, possibly explaining the lack of long term efficacy.

To obtain an optimal training effect, work load must be “shaped” (adjusting the task difficulty to the subjects' performance), and the intensiveness of training should be progressive and challenging. Based on these motor learning principles, Bonzano et al. (15) found in a small pilot RCT that task-oriented upper limb rehabilitation in PwMS had a positive impact on white matter architecture. They demonstrated that connection fibers in the corpus callosum and corticospinal fibers bundles, which are in general affected by the disease, were preserved through active motor training (15). This suggests that upper limb rehabilitation may promote neuroplasticity in MS. Furthermore, the involved tasks should also stimulate subjects' motivation, should be fun, and sustain arousal (16).

With recent technological innovations, such as the development of applications (apps) for mobile phones and/or tablets there are new possible treatment options. The advantages of such apps, certainly build on the premise that these are often mentally stimulating, are game-based, attractive, fun, all aspects which are fundamental to possibly stimulate neuroplasticity in MS. Training with mobile apps may also allow flexible training at home, so PwMS can exercise whenever they want at a convenient time of the day. Recently such an app, called COGNI-TRAck, was developed to train cognition in MS, which was found to be feasible, highly motivating and well-accepted among PwMS (17). A follow-up pilot RCT evaluated the effectiveness of the COGNI-TRAck and demonstrated improved cognitive function, more specifically working memory (18). With respect to dexterity, a first new app has been developed called “Finger Zirkus^©^,” by a team of experts including an occupational therapist (AO, see for acknowledgments), graphic designer, and IT expert. The app is already available to be downloaded from Google Play store or Apple Store (see for more details: www.fingers-in-motion.de). The app specifically contains six dexterous exercises, which are explained more in the methods. The exercises are goal oriented, repetitive, give direct feedback and most importantly are fun to to. To ensure constant improvement, the speed and difficulty of the exercises are progressively adapted to the individuals' performance limit. To our knowledge the efficacy of this tablet app-based training has not been investigated in MS.

The aim of the present study will therefore be to investigate the effectiveness of a tablet app-based dexterity home-based intervention in MS (TAD-MS). The short and long-term benefits of this training program will be compared with a Thera-band® training program. For these purposes, an observer-blinded proof-of-concept RCT will be performed. We hypothesize that TAD-MS will improve dexterity, leading to improved ADL, and HrQoL in PwMS.

## Methods and Design

### Trial Design

**Figure 1:**
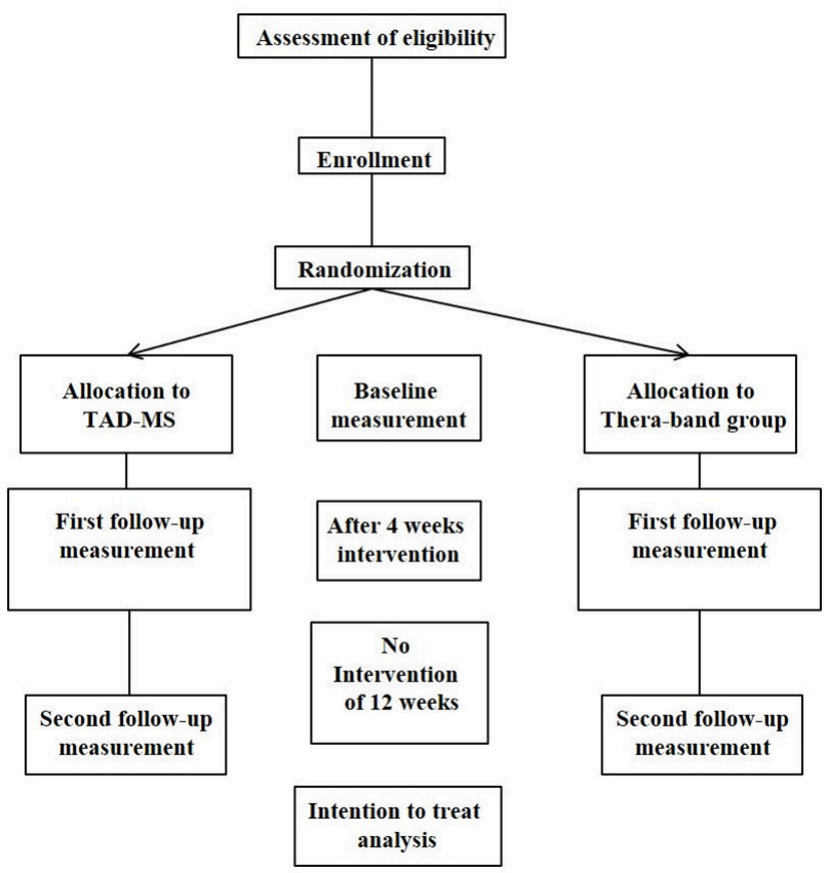
Study flow chart.

An observer-blinded RCT will be performed with random allocation of intervention, with pre- (baseline) and post-intervention measurements, at 4 weeks immediately after intervention and a follow-up 12 weeks later, after finishing the training. The study flow chart is shown in see Figure 1. Ethical approval has been given by the Ethics committee of the State of Luzern (EKNZ/2017-00768), Switzerland. Trial registration is Clinicaltrials.gov NCT03369470 and SNCTP clinical trials registry SNCTP000002451. The study will be performed according to the CONSORT (Consolidated Standards of Reporting Trials) statement, http://www.consort-statement.org/.

### Participants

We aim to recruit 70 PwMS. The inclusion criteria are as follows: males and females, age 18–75, diagnosis of MS (primary or secondary progressive, relapsing-remitting) following the revised McDonald criteria (19). In addition, patients must report difficulties in dexterity that impact ADL and/or have a pathological Nine Hole Peg Test (9HPT) according to cut-off values (20). The exclusion criteria will be other conditions that may limit hand function training or, impaired cognitive functioning (Mini Mental Status Examination score <24).

### Sample Size Calculation

The significance level alpha is defined two-tailed at *p*-value of 0.05 for detecting 20% difference between groups on the Arm Function in Multiple Sclerosis Questionnaire (AMSQ) in favor of the App-based dexterity group. Based on mean expected baseline values of 50.2 ±14.0 (pooled mean and standard deviations values) of the whole group, a 20% difference would reflect a 10-point improvement. Following Cohen's guidelines (21), the probability to prevent type II error is set at 0.20 assuming a 1-beta value of 0.80. The power analysis (clinical superiority trial), with the above-mentioned parameters, resulted in a total sample size of 62 persons, i.e., 31 people per group. Considering a maximal drop-out rate of 10% we aim to recruit 70 patients in total.

### Recruitment

Potential participants will be recruited from the MS center, Luzerner Kantonsspital, Luzern. In addition, Swiss Multiple Sclerosis Society, which is a non-profit patient organization, will be contacted and asked to inform their members about the ongoing study. Prior to study participation, written informed consent will be obtained from all patients according to the latest Declaration of Helsinki.

### Randomization and Blinding

An independent biostatistician (DC, see acknowledgments) will execute a balanced (1:1 ratio) group allocation using a computerized randomization protocol. Two clinicians (JvB, CB), with experience in the field of MS rehabilitation, will be responsible for the assessments and instruction of both interventions. All outcome measures (baseline, post-intervention and follow-up measures) will be videotaped and rated by an independent rater blinded to randomization procedure and group allocation.

### Treatment Interventions

Two home-based treatment conditions are investigated in this study: the experimental condition (TAD-MS) and the control condition (Thera-band®). Patients will receive instructions for their home-based training programs by a trained clinician (JvB, CB). Both groups will train 5 days per week (approximately 30 min per day) for 4 weeks. All exercises will be performed with both hands. Besides therapist instructions, for each exercise the app contains a demonstration video and a text explaining how to perform the exercises. The app is linked with a website in which a clinician can log in and track the patient's performance, so compliance to the protocol can be monitored. Patients exercising with Thera-band® receive a booklet explaining all exercises with pictures and text instructions. This booklet also contains a diary to document if an exercise is performed and, if applicable, the time needed to accomplish the exercise.

#### Tablet App-Based Dexterity Intervention (TAD-MS)

PwMS allocated to the TAD-MS will receive a tablet with the app on it. The app contains six different exercises. During a first training session PwMS receive instructions about the log-in procedure and functionality of the web-interface and exercise app. The therapist can pre-select the exercises for the patient. For more information about TAD-MS a step by step manual is presented in Supplementary File 1.

#### Control Intervention

PwMS allocated to the control group will receive Thera-band-exercises on paper [see (14)]. PwMS will perform seven upper extremity strength-training exercises using a Thera-band®, which are illustrated in detail in Supplementary File 2. As TAD-MS, both arms will be trained equally long.

### Outcome Measures

#### Primary End Point

The AMSQ measures manual dexterity in MS patients and shows good validity, test-retest reliability and inter-observer reliability (22). Recently, a German version of the AMSQ was validated (23). It contains 31 questions on a unidimensional scale that are formulated as “during the past 2 weeks, to what extent has MS limited your ability to …?.” Response categories are “not at all,” “a little,” “moderately,” “quite a lot,” “extremely,” and “no longer able to.” One final sum score is obtained ranging from 0 to 100 with higher scores indicating more dexterous difficulties.

#### Secondary End Points

The 9HPT is a reliable, valid and sensitive test to quantify impaired dexterity, and has been validated in patients with MS (13). While seated at a table, patients have to put nine pegs out of a shallow container into nine holes one at a time and then remove them again one at a time back into the shallow container. The time to perform the task is measured. Age and gender-dependent normal values are available (20).

The Coin rotation Task (CRT) is a sensitive measure to quantify fine coordinated finger movements in MS (9). Participants have to rotate a 20 swiss rappen coin (corresponding to a dime), as quickly as possible between their thumb, index and middle finger. The time to perform 20 half turns is measured twice on both hands and mean values are taken for each hand. We found values of 18.75 s for the dominant and 19.25 s for the non-dominant hand to be optimal cut-offs that are highly sensitive and specific for detecting impaired dexterity (9).

The hand-held JAMAR dynamometer (Sammons Preston Rolyan, 1000 Remington Blvd, Bolingbrook, IL, 60440) is a reliable and valid test to measure isometric grip strength in healthy subjects and PwMS (13, 24). Testing will be performed in a seated position with 90° flexion of the elbow next to the body. Mean values (kilograms force) of three maximum voluntary grip strength movements are taken for each hand.

The Multiple Sclerosis Impact Scale 29 (MSIS-29) is a HrQoL questionnaire assessing the impact of MS on physical and psychological functions (25, 26). It is formed by 29 items: 20 about physical activity and ADL and 9 on psychological status of the person. Each item can be scored with a value from 0 to 5; total score is given by the sum of all the items and is then transformed to a range from 0 to 100. A higher value corresponds to a worse perception of subject's HrQoL.

### Statistical Analysis

Descriptive statistics will be used to present baseline characteristics and results of outcome measurements. The main analysis will be an analysis of covariance (ANCOVA) with between-group factor Intervention (2 levels) and within group factor Time (3 levels). Baseline scores will be used as covariates. Two-sided 95% confidence intervals (CI) will also be calculated, as well as effect size (Cohen's d). *Post-hoc* Tukey test will be used to test for pairwise comparisons. The Benjamini–Hochberg procedure will be applied to control the false discovery rate (27). According to the Intention to treat (ITT) principle every randomized PwMS, including the drop-outs, will be included for final evaluation. Multiple imputation methods will be used to handle missing data, caused by random drop outs. For all analyses the level of significance will be set at *p* < 0.05. Statistical analyses will be performed using PASW for Windows (version 23.0; SPSS, Inc. Chicago, IL).

### Availability of Data and Materials

In accordance with Good Clinical Practice Guidelines members of the ethics committees will be granted access to the original clinical data (coded) for audits or inspections, via the principal investigator. Throughout the entire study, strict confidentiality is ensured. All data will be coded and archived at the Luzerner Kantonsspital throughout the whole study period and archived for 15 years. All measurements (AMSQ, 9HPT, CRT, Jamar, MSIS-29), which are specifically related to the study design, will be entered directly into electronic Case Report Forms (eCRF).

## Discussion

Impaired dexterity is a frequently reported problem in MS, leading to reduced HrQoL (2, 4, 7). However, evidence-based upper limb therapies are still scarce mainly due to lack of methodologically well-conducted trials in this neglected field (4). New technology such as tablet app-based training, has been shown to improve working memory in MS (18).

The aim of the present proof-of-concept RCT is to investigate the effectiveness of a tablet app-based dexterity training at home, compared with a non-dexterity focused Thera-band® program. The exercises within the TAD-MS intervention focus on different components of dexterity such as pinch grip, coordinated finger movements, consequently being highly specific to improve dexterity. In addition, all exercises are game-based and designed to be challenging and to trigger motivation. During the performance of the dexterity exercises the app provides direct feedback of performance which can lead to immediate perception of error and correction of movement, increasing the effectiveness of progressive motor learning training (28, 29). Instead, the Thera-band® program includes non-specific upper limb and hand strength exercises.

The total dosage of the TAD-MS intervention is 600 min of dexterity training (30 min five times a week, 4 weeks), representing a trade-off between feasibility and adequate dosage. Although no studies have investigated tablet-app based dexterity training in MS, a previous home-based sensory re-education training (3 weeks, five times a week 15 min, 300 min total training) induced significant in manual dexterity (30), with stronger effect size when compared with another dexterity interventions (4) Additionally, another home-based dexterity program achieved adequate effect sizes with a 4-week program and 600 min of dexterity training (14). Therefore, the choice of the dosage of our TAD-MS seems appropriate to significantly improve dexterity. The TAD-MS has the potential to be easily implemented in the daily routine of PwMS, since no extensive instruction is needed and the program is not time consuming. Furthermore, TAD-MS is efficient in terms of staff resources as patients can train independently at home without continuous guidance from a therapist. Such cost-effectiveness should be investigated in future studies.

In sum, we expect that the presented tablet app-based program, TAD-MS, through provision of challenging, stimulating dexterity training in the patients' home-environment will be effective in PwMS.

## Dissemination

When protocol amendments are needed (e.g., to include another participating center), ethical approval will be obtained first. After having obtained this approval, relevant adaptations will be made in the relevant clinical trial registry databases.

After completing recruitment, according to the inclusion and exclusion criteria, statistical analyses are planned. When completed, submission to a peer-reviewed journal is planned. In addition, results will be published in patient and healthcare professional magazines and will be presented at national and international congresses.

## Author Contributions

JvB physical therapist, junior researcher and movement scientist, responsible for patient recruitment, provides data collection, manuscript writing. EvW senior researcher and movement scientist, gave critical review of concept and design of the study, and critically revised the manuscript. CB research assistant, responsible for patient recruitment, provides data collection, she critically revised the manuscript. MR senior researcher and movement scientist, gave critical review of concept and design of the study, and critically revised the manuscript. CK neurologist, provided concept and design of the study and critically revised the manuscript. TV is a senior researcher and principal investigator, obtained funding, conceived the idea for the present study, overall project coordination, manuscript writing. All authors contributed to the design of the interventions and outcome measures. All authors assisted in editing and reviewing the submitted manuscript. They all read and approved the final form.

### Conflict of Interest Statement

The authors declare that the research was conducted in the absence of any commercial or financial relationships that could be construed as a potential conflict of interest.

## Abbreviations

Abbreviations: MS: Multiple Sclerosis
ADL: Activities of daily living
RCT: Randomized controlled trial
AMSQ: Arm Function in Multiple Sclerosis Questionnaire
MSIS-29: Multiple Sclerosis Impact Scale 29
9-HPT: Nine-hole peg test
CR: Coin rotation
PT: Physical therapy
HrQoL: Health related Quality of life
CI: confidence interval
ITT, Intention to treat CRF: Case Report Form
PwMS: Patients with Multiple Sclerosis.
