# Supplementary material for: Tablet App Based Dexterity Training in Multiple Sclerosis (TAD-MS): Research Protocol of a Randomized Controlled Trial
Source: Front Neurol. 2019 Feb 11;10:61. doi: 10.3389/fneur.2019.00061 (PMC6378288; doi:10.3389/fneur.2019.00061)
Supplement: Supplementary file 1 [file Data_Sheet_1.docx]

**TAD-MS**

Finger Zirkus© app


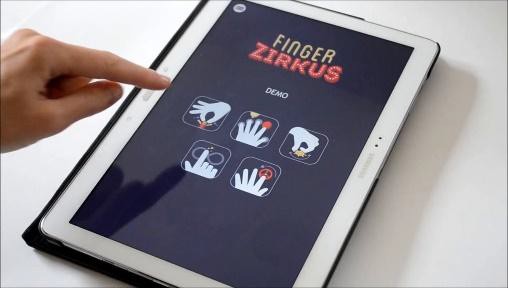


Log into in the app:

After opening the Finger Zirkus© app, two options appear: “PLAY” and “settings”. The patient can choose the “PLAY” button to log into his own secured account and the option “settings” can be used by the clinician to log in for the patient. In order to so, the clinician has to select “settings”, “client documentation”, log in with the four-numbered therapist login and select the corresponding username. The “settings” button can also be used to access the demo exercises.

Calibration:

When using the app for the first time, the clinician has to calibrate the 5 fingers of the patient. By calibrating the patient’s fingers, the app automatically saves the position of each fingertip when the hand is placed flat on the tablet. In this way, all the following exercises are individually adapted to the patient’s hand. The first calibrating is done by selecting “settings”, “client documentation”, logging in with the four-numbered therapist login and selecting the corresponding username. The patient has to place all the fingertips on the tablet screen, while the hand and fingers are relaxed and in a physiological position. The clinician selects “calibrate” when the patient’s hand is in the correct position. Make sure that there is enough space between the fingers and there are five white dots on the screen before selecting “calibrate”. It is possible to recalibrate the patient’s hand whenever it is needed.

The Finger Zirkus© app contains six exercises.

1. Acrobat seesaw:


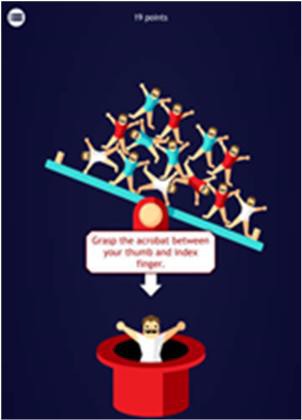


Pinch grip: This grip is important e.g. to pick up small objects (coin, ring) and is needed for writing. The patient has to grasp the acrobat between thumb and index finger, lift the hand off the screen and place the acrobat on the seesaw back on the screen. For every correctly placed acrobat on the seesaw, the patient collects one point. The level of difficulty can be increased by increasing the speed by which the acrobats are placed on the seesaw. There is online visual feedback available during the game. When the pinch grip on the acrobat is executed correctly, the acrobat disappears and when the acrobat is placed correctly on seesaw, the acrobat reappears. When the pinch grip is not or incorrectly executed, a text balloon pops up above the acrobat saying “grasp the acrobat between your thumb and index finger” and when the acrobat is not or incorrectly placed on the seesaw, a text balloon pops up above the seesaw saying “place the acrobat here”.

1. Balloons.


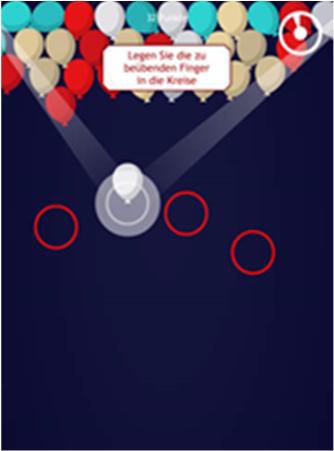


Selective finger tapping: This movement is needed for using the numbers pad or touchscreen on the telephone, ringing the doorbell, or typing on the computer keyboard. The patient has to place all fingers in the white circles which appear on the tablet screen. When a balloon appears underneath a white circle the patient has to lift this finger briefly off the screen and place it back again. All other fingers have to remain on the other white circles. By doing this, the next balloon will pop up. For every correctly finger lift off and on (whilst the other fingertips remain on the other white circles), the patient collects one credit point. The level of difficulty can be increased by increasing the speed in which the fingers should be lifted and placed back. There is online visual feedback available during the game. When a finger is not or incorrectly placed in the white circle, the corresponding circle will turn red and a text balloon pops up above the fingers saying “place your fingers in the circles”.

1. The wheel:


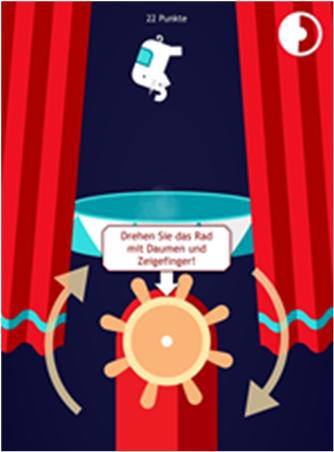


Rotation: This complicated movement requires a combination of rotation and extension/flexion in various joints in fingers, wrist and lower arm. It is needed to screw lids on and off, opening and closing a water-tap and for tightening screws. The patient has to place thumb and index finger on the wheel and turn it clockwise. For every correct wheel turn, the patient collects one credit point. The level of difficulty can be increased by increasing the speed in which the wheel is turned. There is online visual feedback available during the game. When the wheel is turned correctly, the curtains open and

reveal jumping elephants. When the wheel is not or incorrectly turned, the curtains slowly start to close again and a text balloon pops up above the wheel saying “turn the wheel with thumb and index finger”.

1. Horizontal eight:


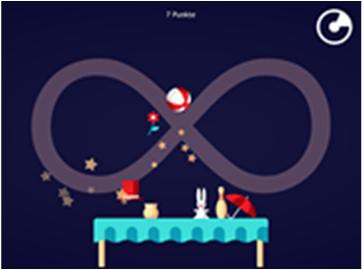


Crossing mid-line: This is an important skill that mimics movements used in everyday life, e.g. tying shoelaces, writing. The patient has to track the horizontal eight with the index finger in a continuous and relaxed movement without interruption. For each correctly traced horizontal eight, the patient collects one credit point. The level of difficulty can be increased by increasing the speed in which the horizontal eight should be traced. There is online visual feedback available during the game. When the horizontal eight is correctly traced, a trail of stars will follow the index finger and one object will start to lift off from the table for each completed horizontal eight. When the index finger does not follow the horizontal eight correctly, the path will turn red. When the index finger completely leaves the horizontal eight or the screen, all objects which were lifted off, will fall down and a text balloon pops up above the horizontal eight saying “trace the path”.

1. Elevator:


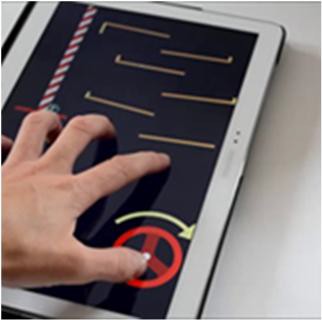


Selective finger rotation I. This movement is needed, in combination with others, for many activities, e.g. applying cream or make-up to ones face, buttoning up, cleaning glasses and manipulating a fork. The patient has to turn the wheel using the thumb by doing a circular motion. The other fingers should be placed in the calibrated white circles and stay in place. For every three correct wheel rotations, the patient collects one credit point. The level of difficulty can be increased by increasing the speed in which the wheel is turned by the thumb. There is online visual feedback available during the game. When the wheel is turned correctly, whilst the other fingers remain in place, the elevator goes up. When the wheel is not or incorrectly turned, or the other fingers do not remain in place, the elevator does not move and a text balloon pops up above the fingers saying “place your fingers in the circles”.

1. Elevator: Selective finger rotation II. The patient has to turn the wheel using the index finger by doing a circular motion. The other fingers should be placed in the white circles and should remain in place. For every three correct wheel rotations, the patient collects one credit point. The level of difficulty can be increased by increasing the speed in which the wheel is turned by the index finger. There is online visual feedback available during the game. When the wheel is turned correctly whilst the other fingers remain in place, the elevator goes up. When the wheel is not or incorrectly turned or the other fingers do not stay in place, the elevator does not work and a text balloon pops up above the fingers saying “place your fingers in the circles”.

During each exercise there is a 1-minute clock displayed in the upper right corner and the number of collected credit points is displayed in the upper middle of the screen. The sequence of exercises will automatically load, starting with the first exercise “Acrobat seesaw: Pinch grip”. The patient can select “instructions” to read what to do during the exercise or select “PLAY” when no instructions are further needed. After selecting “PLAY” the exercise will take 1 min and afterwards the collected amount of credit points is displayed.

After all exercises are done, the app will display a summary of all exercises and the corresponding collected credit points.

All pictures are used with permission from © fingers in motion, 2019.
