# Supplementary material for: Tablet App Based Dexterity Training in Multiple Sclerosis (TAD-MS): Research Protocol of a Randomized Controlled Trial
Source: Front Neurol. 2019 Feb 11;10:61. doi: 10.3389/fneur.2019.00061 (PMC6378288; doi:10.3389/fneur.2019.00061)
Supplement: Supplementary file 2 [file Data_Sheet_2.docx]

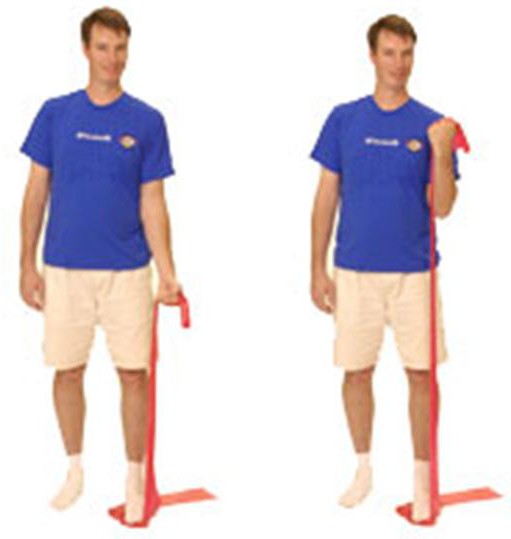


# Starting position:

Standing upright, Thera-band® fixated underneath the foot

**Execution:** Hand is supinated, whilst bending the elbow Repeat this exercise 5 times

# 2. Elbow extension


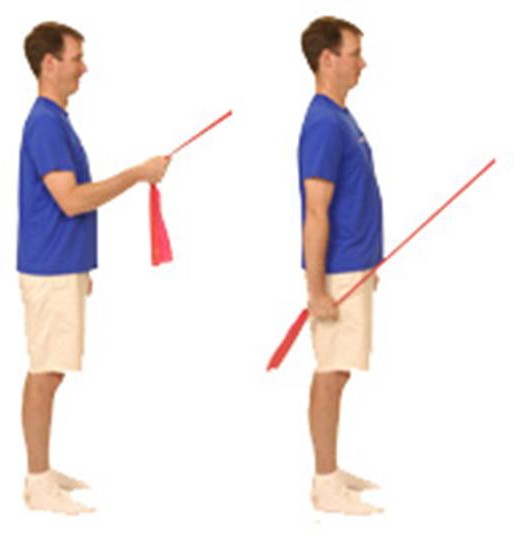


**Starting position:**

Standing upright. Thera-band® fixated at the top of a door. **Execution:** Stretching the elbow Repeat this exercise 5 times


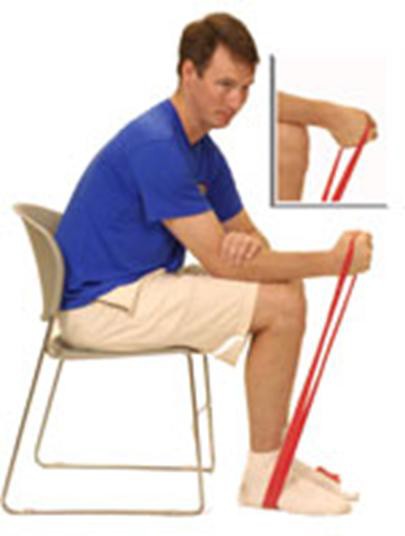


# Starting position:

Seated position. Forearm is fixated on the thigh with opposite hand. Thera-band® is fixated under the foot. Hand in neutral position

**Execution:** Abduction of the wrist. Repeat this exercise 5 times

# 4. Hand pro-supination


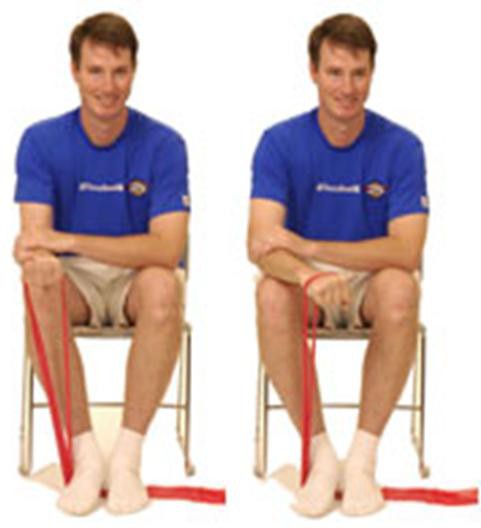

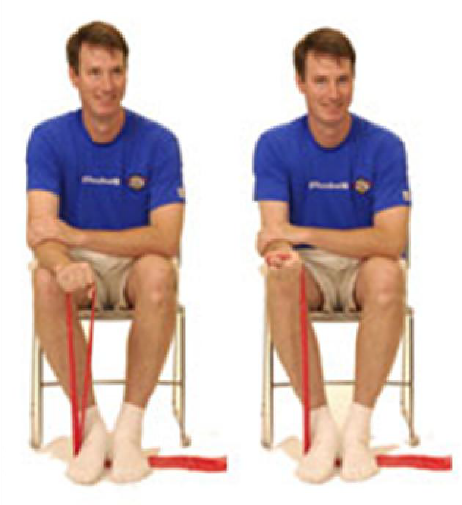


**Starting position:**

Seated position. Forearm fixated on the thigh with opposite hand. Thera-band® fixated under the foot.

**Execution:** Hand is 180° pronated and 180° supinated. Repeat this exercise 5 times


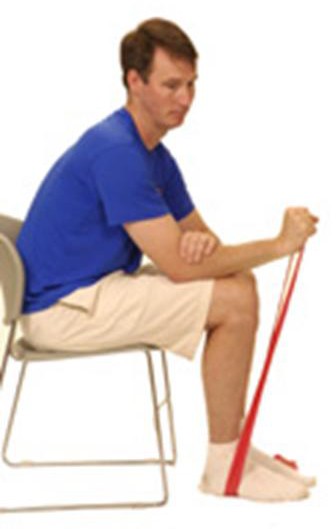


# Starting position:

Seated position. Forearm fixated on the thigh with opposite hand. Thera-band® fixated under the foot. Hand is pronated

**Execution:** Extending the hand. Repeat this exercise 5 times

# 6. Hand flexion


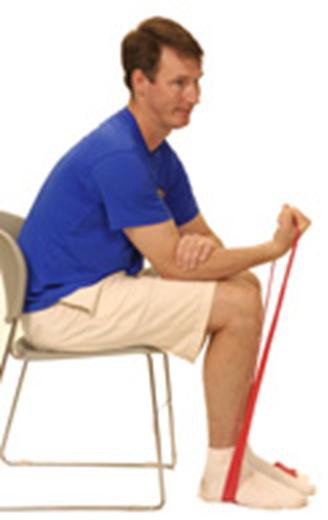


**Starting position:**

Seated position. Forearm fixated on the thigh with opposite hand. Thera-band® fixated under the foot. Hand is supinated

**Execution:** Flexion of the hand. Repeat this exercise 5 times
